# Supplementary material for: Frequent miRNA-convergent fusion gene events in breast cancer
Source: Nat Commun. 2017 Oct 5;8:788. doi: 10.1038/s41467-017-01176-1 (PMC5629207; doi:10.1038/s41467-017-01176-1)
Supplement: Supplementary file 1 — Supplementary Information [file 41467_2017_1176_MOESM1_ESM.pdf]

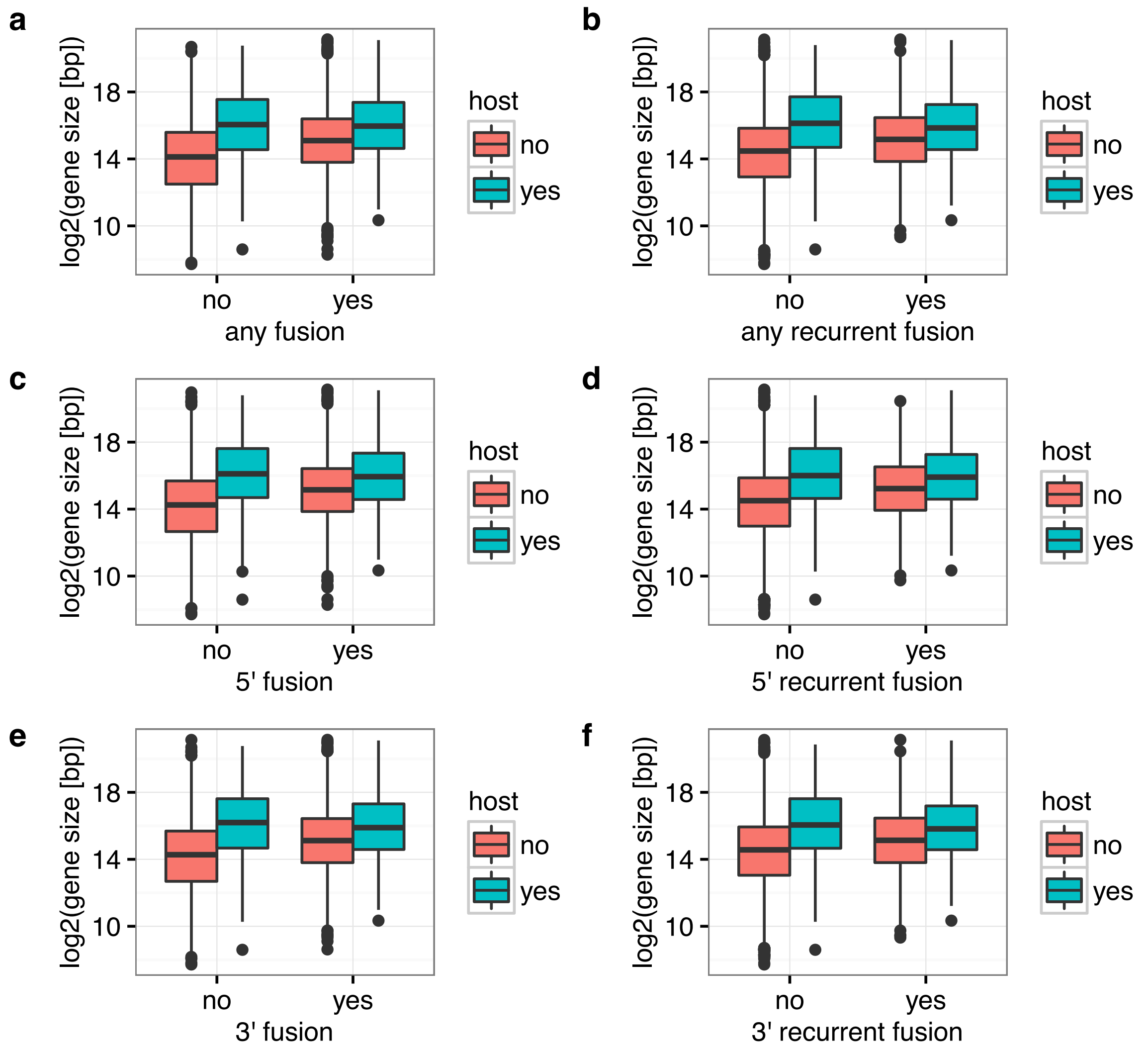

Supplementary Figure 1. Both miRNA host genes and fusion gene partners tend to be longer than other genes, but there was no significant size difference between miRNA hosts with or without fusions. All fusion partners are included in (**a-b**), 5' partners in (**c-d**), and 3' partners in (**e-f**). Panels (**a,c,e**) show all fusions, while only fusions recurrent in at least three tumors with the host gene in the same position are shown in (**b,d,f**).

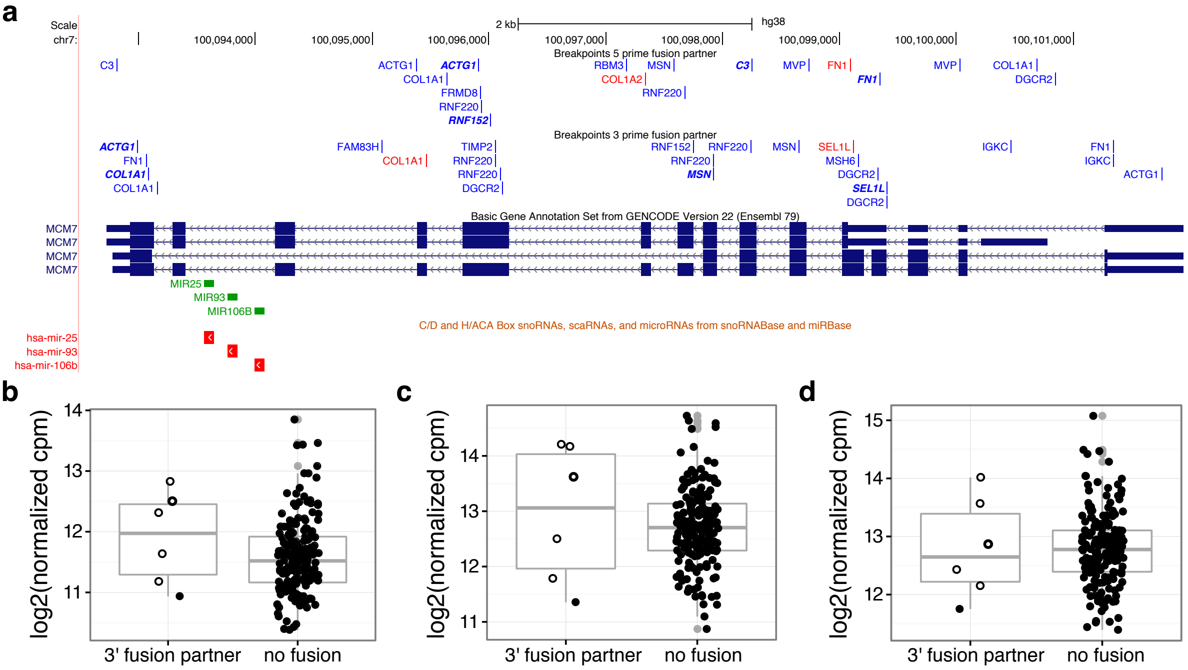

Supplementary Figure 2. Breakpoints and fusion partners for 5' and 3' *MCM7* fusion transcripts (**a**). Red marks in-frame fusions between partner gene coding sequences (CDSs) and blue other fusion transcripts with out-of-frame fusions between partner gene CDSs in bold italic font. Expression of mature miRNAs in the *mir-106b/93/25* cluster showed a bimodal distribution in the 6 tumors with 3' *MCM7* fusions and available miRNA expression data, and there was significant overexpression of miR-25-3p (**b**), but not miR-93-5p (**c**), or miR-106b-5p (**d**) compared with tumors without *MCM7* fusions. Samples with *MCM7* fusions are shown as filled circles for in-frame fusions between partner gene CDSs, open circles with thick line for out-of-frame fusions between partner gene CDSs, and open circles with thin line for all other fusion transcripts.

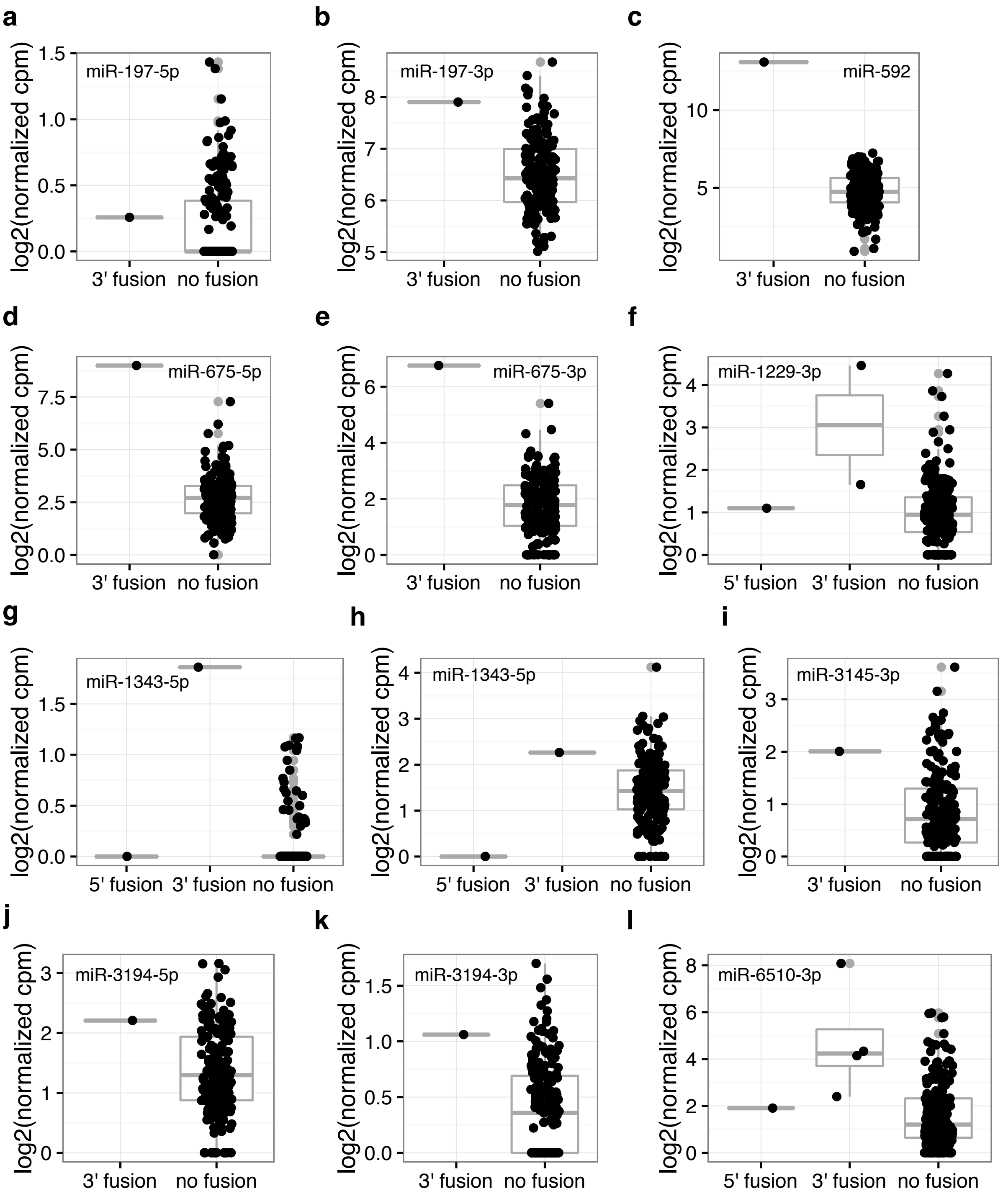

Supplementary Figure 3. Examples of miRNAs upregulated in tumors with fusion transcripts involving their host gene as 3' partner compared with tumors without host gene fusions. The expression in samples that exclusively have fusion transcripts with the miRNA host gene as 5' partner is also shown when present. Expression is shown as $\log_2$ of TMM-normalized counts per million reads (cpm).

a

b

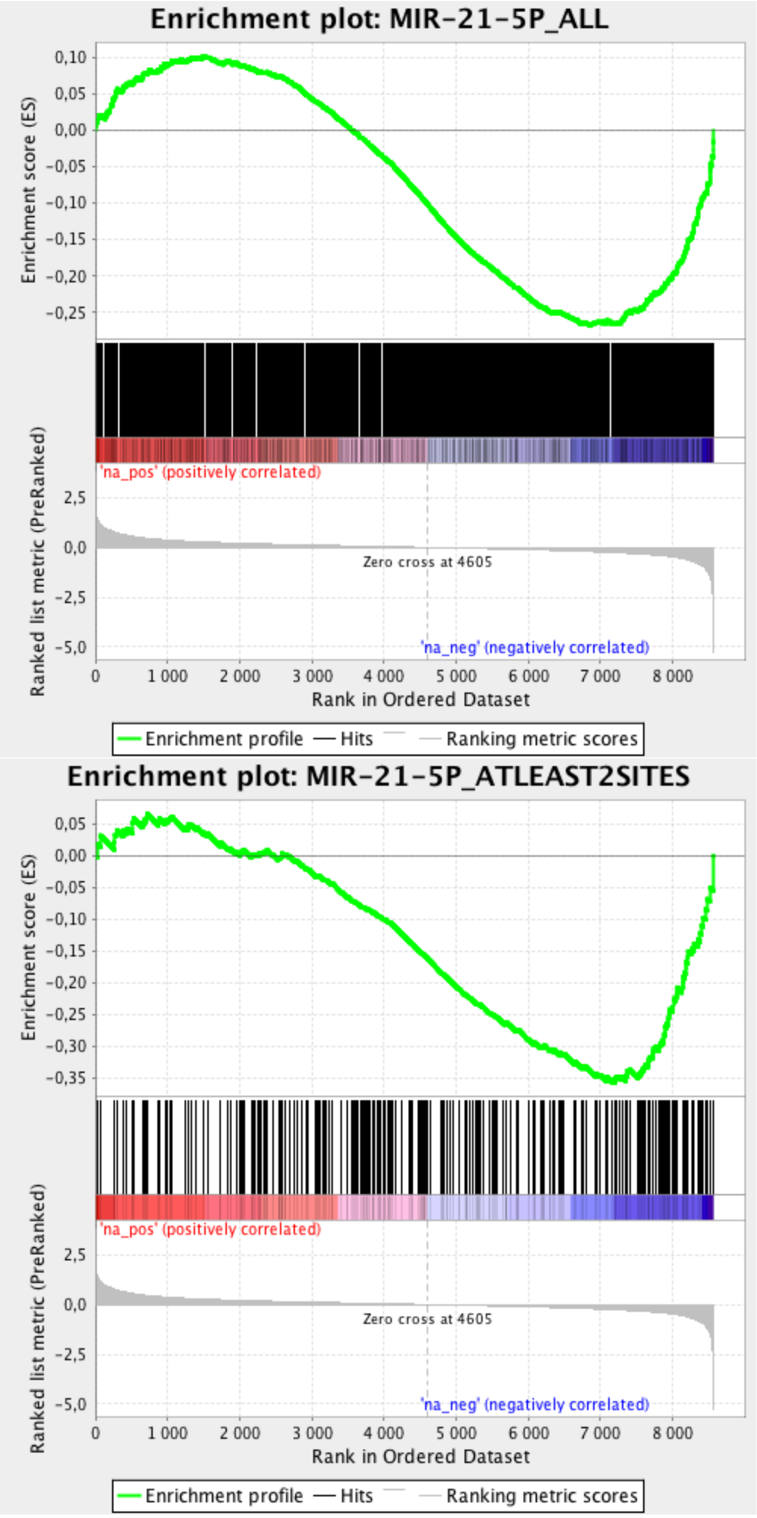

Supplementary Figure 4. Predicted targets for miR-21-5p were enriched among genes downregulated in tumors with *VMP1* fusion transcripts including *mir-21* vs tumors without *mir-21* fusions in a gene set enrichment analysis (GSEA). The FDR q-value was < 0.001 for all targets for miR-21-5p predicted by TargetScan 7.1 (**a**) and 0.026 for targets with at least 2 predicted target sites (**b**).

Supplementary Table 1. Fusion transcript classification according to FusionCatcher for all fusion transcripts, fusion transcripts including a miRNA within any partner gene, and transcripts including a miRNA within the 5' or 3' fusion partner, respectively.

class	all_transcripts	miRNA_incl	5prime_incl_miRNA	3prime_incl_miRNA
in-frame	32313	5454	4037	1632
out-of-frame	39194	6436	4799	1877
---/CDS(complete)	15	13	12	1
---/CDS(no-known-start-or-end)	29	4	4	0
---/CDS(truncated)	16615	9914	9411	688
---/UTR	11054	8274	8170	143
---/exonic(no-known-CDS)	55	32	29	7
---/intronic	2553	960	721	313
CDS(complete)/---	8	1	1	0
CDS(complete)/CDS(no-known-start-or-end)	1	1	1	0
CDS(complete)/UTR	136	29	22	9
CDS(complete)/exonic(no-known-CDS)	11	1	1	0
CDS(complete)/intergenic	2	2	2	0
CDS(complete)/intronic	159	48	24	28
CDS(no-known-start-or-end)/---	13	2	1	1
CDS(no-known-start-or-end)/CDS(complete)	11	3	3	0
CDS(no-known-start-or-end)/CDS(truncated)	415	52	19	33
CDS(no-known-start-or-end)/UTR	170	11	8	3
CDS(no-known-start-or-end)/exonic(no-known-CDS)	10	0	0	0
CDS(no-known-start-or-end)/intronic	210	18	4	14
CDS(truncated)/---	12643	450	236	242
CDS(truncated)/CDS(no-known-start-or-end)	420	59	53	6
CDS(truncated)/UTR	35945	5444	4474	1110
CDS(truncated)/exonic(no-known-CDS)	1723	189	124	73
CDS(truncated)/intergenic	38	11	3	11
CDS(truncated)/intronic	37113	9141	4078	5713
UTR/---	2260	212	120	98
UTR/CDS(complete)	169	18	14	4
UTR/CDS(no-known-start-or-end)	178	14	11	3
UTR/CDS(truncated)	38034	4766	3028	1887
UTR/UTR	23541	2612	2002	652
UTR/exonic(no-known-CDS)	788	72	38	45
UTR/intergenic	33	14	1	13
UTR/intronic	26205	5262	2067	3509
exonic(no-known-CDS)/---	17	0	0	0
exonic(no-known-CDS)/CDS(complete)	3	0	0	0
exonic(no-known-CDS)/CDS(no-known-start-or-end)	4	0	0	0
exonic(no-known-CDS)/CDS(truncated)	1287	98	39	61
exonic(no-known-CDS)/UTR	943	23	4	19
exonic(no-known-CDS)/exonic(no-known-CDS)	52	4	4	0
exonic(no-known-CDS)/intergenic	1	0	0	0
exonic(no-known-CDS)/intronic	861	138	4	135
intergenic/---	1	0	0	0
intergenic/CDS(complete)	3	1	1	1
intergenic/CDS(no-known-start-or-end)	4	0	0	0
intergenic/CDS(truncated)	194	21	8	13
intergenic/UTR	598	126	81	51
intergenic/exonic(no-known-CDS)	6	1	1	0
intergenic/intronic	73	19	10	11
intronic/---	1412	166	81	88
intronic/CDS(complete)	243	14	11	3
intronic/CDS(no-known-start-or-end)	258	45	43	2
intronic/CDS(truncated)	72788	10847	7936	3399
intronic/UTR	38223	4747	3921	909
intronic/exonic(no-known-CDS)	1091	205	109	121
intronic/intergenic	19	6	4	4
intronic/intronic	13017	3278	1666	1807
Total:	413162	79258	57441	24739

Supplementary Table 2. Number of genes in recurrent and non-recurrent fusion transcripts containing miRNAs with the corresponding percentage of all unique genes in parenthesis. 5' or 3' fusion partner refers to the position of the gene and recurrent fusions were defined as occurring in at least three tumors with the gene in the same position.

	**any fusion**	**5' fusion partner**	**3' fusion partner**
≥ 1 tumor	11424	9531	9798
≥ 1 tumour and contain miRNA	667 (6%)	567 (6%)	523 (5%)
recurrent (≥ 3 tumors)	6698 (59%)	5316 (56%)	4943 (50%)
recurrent and contain miRNA	446 (4%)	339 (4%)	293 (4%)

Supplementary Table 3. MicroRNAs with increased expression in samples with 3' fusions including the miRNA compared to samples without any host gene fusions. Expression is shown as median counts per million reads (cpm). Cut-offs were median expression in 3' fusion samples ≥ 3 cpm and log2 fold change ≥ 0.30. The significance of differences was tested using Student's t-test for log2(cpm) and p-values corrected for multiple testing by Bonferroni correction. n.s. = not significant, n.t. = not tested (< 3 samples).

miRNA	cpm 3' fusion	cpm no fusion	log2 fold change	Bonferroni corr *p*
hsa-miR-21-3p	4126	1313	1,65	9,91E-08
hsa-miR-21-5p	328611	168358	0,96	7,39E-05
hsa-miR-25-3p	4265	3413	0,32	n.s.
hsa-miR-25-5p	19	11	0,78	n.s.
hsa-miR-26a-1-3p	7	6	0,41	n.s.
hsa-miR-26b-3p	13	10	0,34	n.t.
hsa-miR-33b-3p	31	25	0,30	n.s.
hsa-miR-33b-5p	87	29	1,58	2,22E-03
hsa-miR-93-3p	224	154	0,53	n.s.
hsa-miR-93-5p	10308	7573	0,44	n.s.
hsa-miR-151a-3p	1227	460	1,41	3,14E-02
hsa-miR-151a-5p	218	122	0,84	n.s.
hsa-miR-197-3p	238	102	1,23	n.t.
hsa-miR-340-5p	1091	849	0,36	n.s.
hsa-miR-342-3p	9522	4092	1,22	n.s.
hsa-miR-342-5p	211	79	1,42	n.s.
hsa-miR-483-3p	9	6	0,52	n.s.
hsa-miR-548ah-5p	5	1	2,11	n.t.
hsa-miR-548ao-5p	5	1	3,11	n.t.
hsa-miR-592	8746	36	7,94	n.t.
hsa-miR-629-3p	8	6	0,50	n.t.
hsa-miR-641	12	7	0,79	n.s.
hsa-miR-675-3p	107	3	4,98	n.t.
hsa-miR-675-5p	509	8	5,95	n.t.
hsa-miR-1229-3p	12	1	3,03	6,38E-03
hsa-miR-1249-3p	19	10	0,96	n.s.
hsa-miR-1343-3p	4	2	0,90	n.t.
hsa-miR-3145-3p	3	1	1,39	n.t.
hsa-miR-3194-5p	4	2	0,94	n.t.
hsa-miR-3616-3p	11	2	2,45	n.t.
hsa-miR-3616-5p	12	1	3,77	n.t.
hsa-miR-3909	12	8	0,61	n.s.
hsa-miR-4714-3p	7	1	3,73	4,34E-02
hsa-miR-4728-3p	21	3	3,01	< 2,20E-16
hsa-miR-4802-3p	3	2	0,54	n.t.
hsa-miR-4802-5p	10	7	0,47	n.t.
hsa-miR-6510-3p	77	5	4,03	6,17E-04
