# Supplementary material for: Frequent miRNA-convergent fusion gene events in breast cancer
Source: Nat Commun. 2017 Oct 5;8:788. doi: 10.1038/s41467-017-01176-1 (PMC5629207; doi:10.1038/s41467-017-01176-1)
Supplement: Supplementary file 3 — Description of Additional Supplementary Files [file 41467_2017_1176_MOESM3_ESM.pdf]

Description of Additional Supplementary Files

File Name: Supplementary Data 1
Description: Human miRNAs (miRBase v21) with host gene annotation (GENCODE v22) and fusion transcripts identified in our set of 1553 breast tumors. The total number of detected fusion transcripts across all tumors is given within parenthesis for each host gene-fusion partner pair. The table also shows the overlap with miRNAs found in fusion transcripts from the TCGA Fusion Gene Data Portal and FusionCancer database. Since the Mitelman Database of Chromosome Aberrations and Gene Fusions in Cancer does not contain genomic breakpoint coordinates, this column refers to fusion genes affecting miRNA host genes, without information on whether the corresponding miRNA was included in the fused gene segments.

File Name: Supplementary Data 2
Description: Human miRNAs (miRBase v21) with host gene annotation (Entrez) and fusion transcripts identified in the TCGA data by Yoshihara et al. (Oncogene 2015;34(37):4845-54). The total number of detected fusion transcripts across all tumors is given within parenthesis for each host gene-fusion partner pair.

File Name: Supplementary Data 3
Description: Enrichment of miRNA host genes in fusions is not associated with the protein function of the host (top 5 terms showed).

File Name: Supplementary Data 4
Description: Summary of FusionCatcher output for predicted SREBF1 fusion transcripts. Fusions marked 'readthrough' werenot used for further analysis. Please see the online FusionCatcher documentation at https://github.com/ndaniel/fusioncatcher/blob/master/doc/manual.md for further details regarding the output format.

File Name: Supplementary Data 5
Description: Summary of FusionCatcher output for predicted MCM7 fusion transcripts. Please see the online FusionCatcher documentation at https://github.com/ndaniel/fusioncatcher/blob/master/doc/manual.md for further details regarding the output format.

File Name: Supplementary Data 6
Description: Summary of FusionCatcher output for predicted VMP1 fusion transcripts. The CLTC-VMP1 fusion marked as "readthrough" was not used for further analysis. Please see the online FusionCatcher documentation at https://github.com/ndaniel/fusioncatcher/blob/master/doc/manual.md for further details regarding the output format.
